# Recurrent Acute Kidney Injury Due to Atheroembolic Renal Disease: Diagnostic Utility of Fundoscopy

**DOI:** 10.7759/cureus.91523

**Published:** 2025-09-03

**Authors:** Shiyun Tan, Nigel Fong, Cai Jiashen, Debajyoti Roy

**Affiliations:** 1 Renal Medicine, Changi General Hospital, Singapore, SGP; 2 Renal Medicine, Singapore General Hospital, Singapore, SGP

**Keywords:** aki (acute kidney injury), aki outcome, atheroembolic renal disease, funduscopy, hollenhorst plaque

## Abstract

Atheroembolic renal disease (AERD) is a rare and often underdiagnosed cause of acute kidney injury (AKI), particularly in patients with atherosclerotic disease. We present the case of a 66-year-old man with hypertension, hyperlipidemia, and stage 3 chronic kidney disease (CKD) (baseline creatinine 100-120 μmol/L) who developed AKI after coronary artery bypass graft (CABG) for non-ST-elevation myocardial infarction (NSTEMI), followed by recurrent episodes and progressive renal decline despite no further invasive procedures. Fundoscopic examination revealed Hollenhorst plaques, leading to a clinical diagnosis of AERD. This case underscores the diagnostic value of noninvasive ophthalmologic evaluation in patients at high risk for cholesterol embolism. The patient was managed with supportive measures, including hydration and blood pressure control, which helped stabilize renal function (creatinine improved from 591 to 410 μmol/L). Our objective is to highlight the diagnostic utility of fundoscopy in evaluating unexplained renal deterioration and systemic ischemic symptoms in frail patients where biopsy is contraindicated.

## Introduction

Atheroembolic renal disease (AERD) is an underrecognized cause of acute kidney injury (AKI) resulting from the occlusion of small renal arteries by cholesterol crystal emboli dislodged from ulcerated atherosclerotic plaques [1].

The epidemiology of AERD is difficult to ascertain due to frequent subclinical presentation and underdiagnosis. Reported prevalence rates among patients with atherosclerosis vary widely (e.g., 0.79-10% in different studies), a disparity largely attributable to differing diagnostic criteria (clinical vs. histopathological) and the characteristics of the study populations (e.g., general hospital admissions vs. high-risk cohorts post-vascular intervention) [2,3]. It predominantly affects elderly males with widespread atherosclerosis. While it is classically iatrogenic, triggered by vascular procedures, angiography, or anticoagulation, spontaneous atheroembolism can also occur [4].

The clinical relevance of AERD lies in its diagnostic challenge; it often presents with a non-specific, stepwise decline in renal function and can mimic other causes of AKI. Systemic findings such as livedo reticularis, digital ischemia ("blue toe syndrome"), eosinophilia, and retinal cholesterol plaques (Hollenhorst plaques) can provide crucial diagnostic clues [5,6]. Recognition is critical, as management is primarily supportive, focusing on risk factor modification and avoiding further insults. The prognosis is generally poor, with a significant proportion of patients progressing to end-stage renal disease [7,8]. This case highlights the diagnostic utility of fundoscopy in identifying AERD in a high-risk patient with recurrent AKI, where renal biopsy was contraindicated.

## Case presentation

A 66-year-old man with hypertension, hyperlipidemia, and stage 3 chronic kidney disease (CKD) (baseline creatinine 100-120 μmol/L) developed an initial episode of AKI following coronary artery bypass grafting (CABG) for non-ST-elevation myocardial infarction (NSTEMI), and subsequently experienced recurrent episodes of AKI with progressive decline in renal function, in the absence of further invasive interventions.

**Figure 1:**
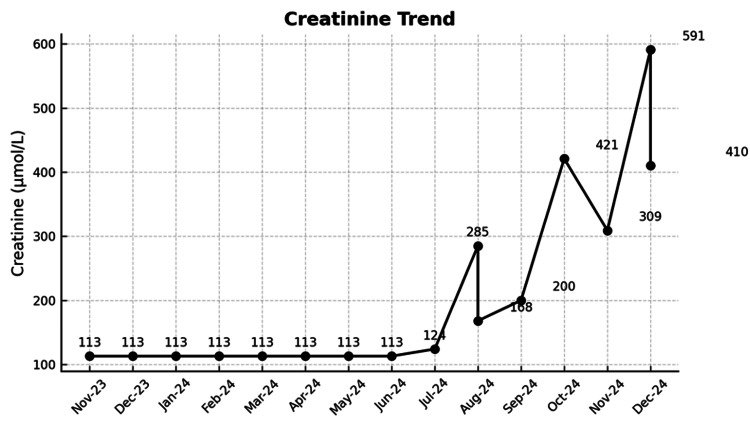
Timeline of serum creatinine values (umol/L) and key clinical events Serum creatinine values are shown over time, illustrating recurrent acute kidney injury (AKI) episodes after coronary artery bypass graft (CABG) surgery and the eventual diagnosis of atheroembolic renal disease (AERD). The figure demonstrates the progression from a stable baseline to recurrent AKI and establishment of a new chronic kidney disease (CKD) baseline.

**Figure 2:**
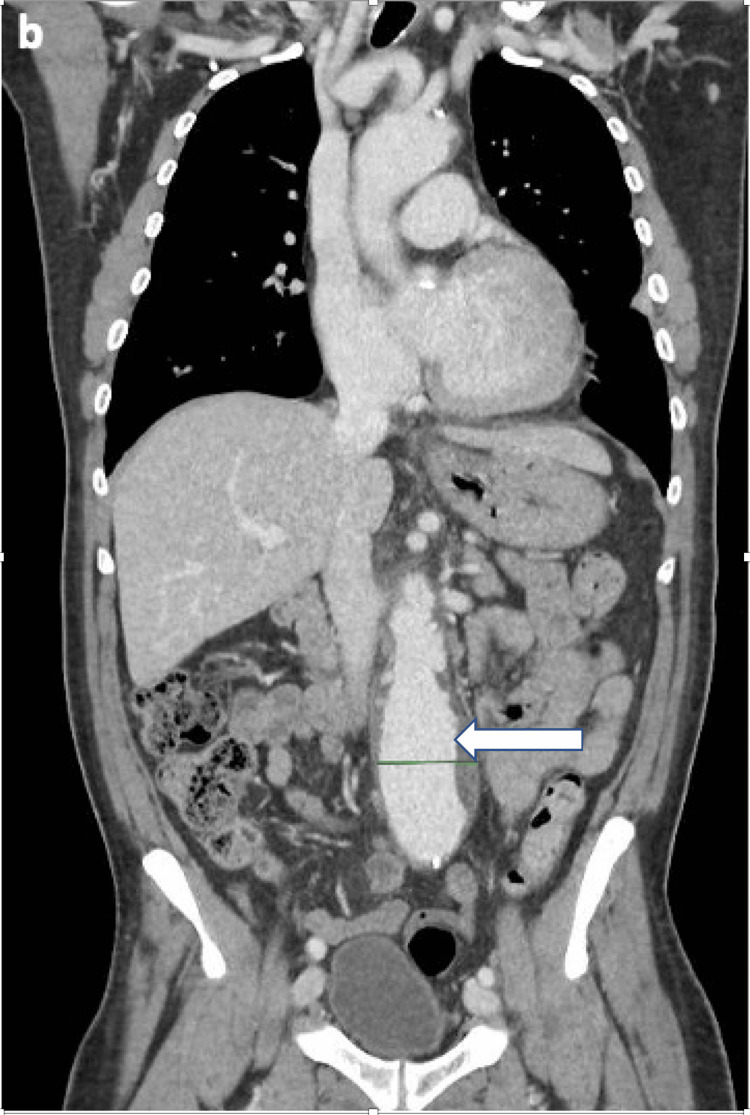
Axial CT aortogram image This single axial image from the November 2024 study shows the infrarenal abdominal aortic aneurysm (AAA). The white arrow indicates the large, concentric mural thrombus. The complex nature of the thrombus and vessel wall is consistent with the presence of atheromatous disease and penetrating ulcers as described in the formal radiology report, which are a known source for spontaneous cholesterol embolization. The lumen of the aneurysm is enhanced by intravascular contrast.

He was first readmitted two months post-CABG with AKI (creatinine 285 μmol/L). Two months later, he presented again with worsening AKI (creatinine 421 μmol/L) and bilateral toe discoloration (Figure 1). A CT scan revealed an infrarenal abdominal aortic aneurysm (AAA) with mural thrombus (Figure 2). Given the significant size of the thrombus and the high associated risk of arterial thromboembolism, apixaban was initiated after multidisciplinary discussion with vascular surgery, weighing the imminent thrombotic risk against the potential for embolization. He was started on apixaban.

**Figure 3:**
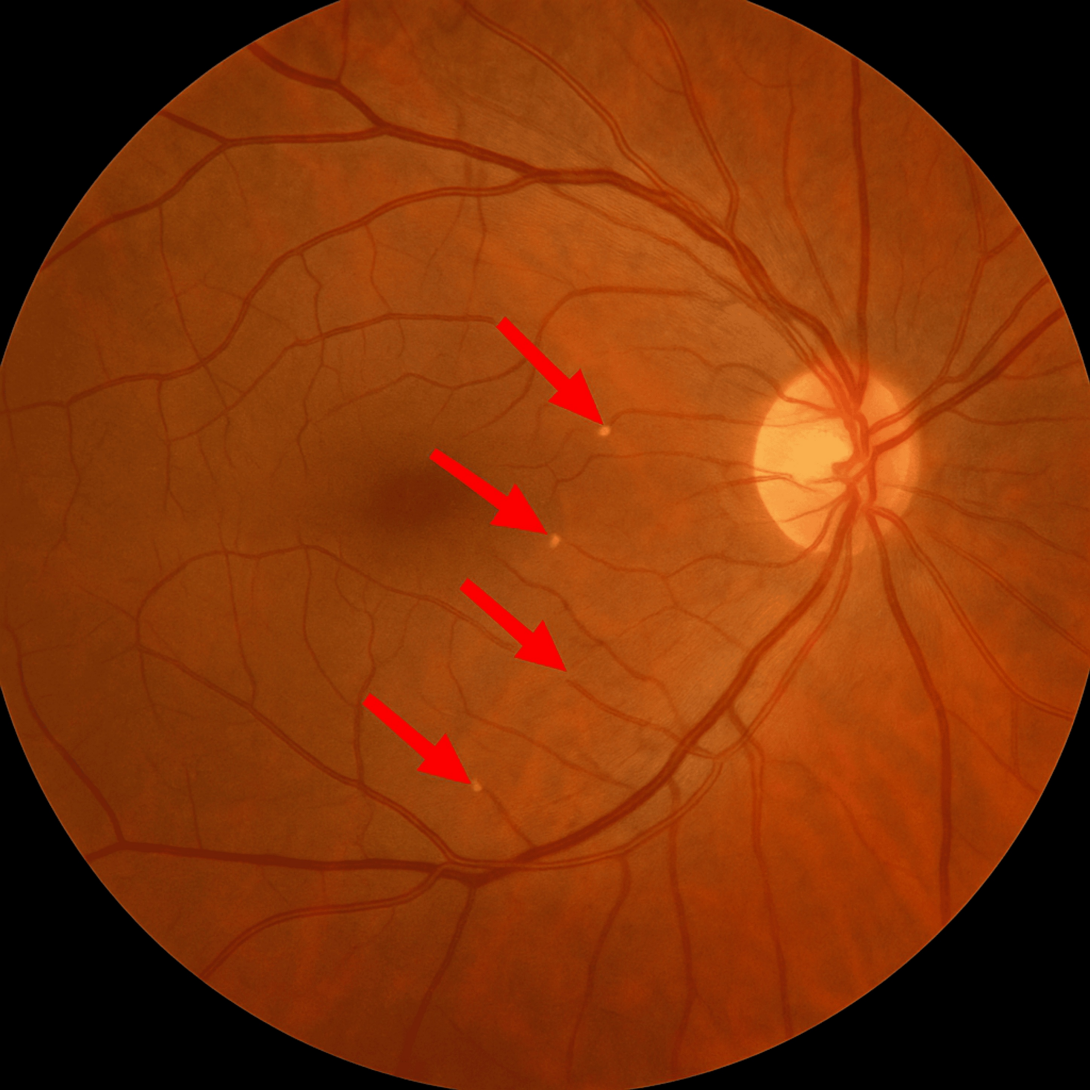
Fundus photograph demonstrating Hollenhorst plaques This fundus photograph shows refractile, yellow-gold cholesterol emboli (Hollenhorst plaques, indicated by arrows) located at the bifurcations of retinal arterioles. This finding suggests ophthalmologic evidence of systemic atheroembolism.

A third episode of severe AKI (creatinine 591 μmol/L) occurred one month after that, accompanied by anemia and abdominal pain. A CT aortogram showed an enlarging AAA with penetrating ulcers. Fundoscopic examination revealed Hollenhorst plaques, confirming the diagnosis of AERD (Figure 3). Upon this diagnosis, apixaban was discontinued to prevent further cholesterol crystal embolization.

Renal biopsy was deferred due to frailty. After a systematic workup excluded other causes of AKI, he was managed supportively. His renal function subsequently stabilized (creatinine 410 μmol/L).

Urine studies were bland (protein-creatinine ratio 1.06 g/g), with borderline eosinophilia. Alternative causes of AKI were systematically evaluated. Pre-renal AKI was unlikely given stable hemodynamics and no nephrotoxic exposures. Post-renal obstruction was excluded by imaging, which showed no hydronephrosis. Glomerulonephritis and vasculitis were considered, but urinalysis revealed bland sediment with minimal proteinuria, and serologies were not supportive. Drug-induced nephrotoxicity was also reviewed, but there was no temporal association.

He was managed supportively with hydration, renal-protective measures, and transfusions for a bleeding gastric ulcer. Renal function stabilized at creatinine 410 μmol/L (estimated glomerular filtration rate (eGFR) 13 mL/min/1.73 m²), representing progression to stage 4 CKD. This level remained stable on subsequent outpatient checks, confirming the cessation of acute embolic activity and a transition to chronic disease management.

## Discussion

A renal biopsy demonstrating cholesterol clefts within arterioles remains the histopathological gold standard for a definitive diagnosis of AERD [5-7]. However, in clinical practice, an invasive biopsy is often contraindicated in frail, high-risk patients due to coagulopathy or overall morbidity. In such cases, a presumptive clinical diagnosis is justified based on the constellation of findings, including unexplained AKI in a patient with advanced atherosclerosis, characteristic peripheral signs (e.g., livedo reticularis, digital ischemia), laboratory abnormalities (e.g., eosinophilia, hypocomplementemia), and supportive ophthalmologic evidence such as Hollenhorst plaques, as demonstrated in this case [7,8]. Our patient exemplifies this scenario, where fundoscopy provided a valuable, non-invasive means of supporting the clinical diagnosis. AERD carries a poor prognosis, with historical data indicating that up to 50% of patients progress to end-stage renal disease requiring dialysis [6,7]. Our patient's course, stabilizing at a new baseline of stage 4 CKD (creatinine 410 μmol/L), exemplifies this guarded outlook; while he avoided dialysis in the short term, his significant residual renal impairment highlights the typically irreversible nature of this injury. This poor prognosis underscores the importance of management being primarily supportive and focusing on halting further progression through statins, meticulous blood pressure control, and, as was critical in this case, the cessation of anticoagulants where feasible.

Given the lack of definitive therapy, treatment strategies like corticosteroids remain controversial. Their use is supported by inconsistent evidence and a lack of randomized controlled trials, with conflicting findings likely stemming from heterogeneity in study design, patient selection, and dosage regimens [9-12]. For our patient, who lacked a pronounced inflammatory phenotype (e.g., absence of fever, significant myalgias, or marked eosinophilia) and whose renal failure was subacute rather than fulminant, the risks of corticosteroid therapy were deemed to outweigh any potential benefit. However, some case series suggest a potential role for a brief, tapered course in a specific subset of patients, particularly those with severe systemic inflammatory manifestations or rapidly progressive renal failure [10,12]. Therefore, the decision to initiate therapy must be highly individualized, weighing the tenuous potential for stabilizing renal function against the risks of infection and other complications in an often frail patient population.

Limitations

This case lacked histopathological confirmation due to the patient's frailty and bleeding risk. Although clinical and fundoscopic findings were consistent with AERD, the absence of a renal biopsy remains a diagnostic limitation. Additionally, long-term renal outcomes and follow-up imaging were unavailable at the time of reporting.

## Conclusions

In high-risk patients with unexplained AKI and systemic features, fundoscopy provides a rapid, noninvasive tool to identify Hollenhorst plaques, which serve as a valuable surrogate marker for systemic atheroembolism. As demonstrated in this case, this finding can strongly support a clinical diagnosis of AERD, particularly when renal biopsy is contraindicated due to patient frailty. While management remains primarily supportive, early diagnosis allows for the crucial withdrawal of precipitating factors, such as anticoagulation, and the initiation of renal-protective measures, which may help to stabilize function and alter the course of the disease. Thus, a simple fundoscopic examination can be a pivotal first step in diagnosing this challenging condition and guiding management to prevent further iatrogenic harm.
